# Глубокая интронная мутация в гене AR как причина синдрома резистентности к андрогенам: сложности диагностики

**DOI:** 10.14341/probl12799

**Published:** 2021-09-19

**Authors:** Н. Ю. Калинченко, В. М. Петров, А. В. Панова, А. Н. Тюльпаков

**Affiliations:** Национальный медицинский исследовательский центр эндокринологии; Национальный медицинский исследовательский центр эндокринологии; Национальный медицинский исследовательский центр эндокринологии; Институт общей генетики им. Н.И. Вавилова РАН; Медико-генетический научный центр им. академика Н.П. Бочкова; Республиканская детская клиническая больница

**Keywords:** рецептор к андрогенам, резистентность к андрогенам, нарушение формирования пола, аберрантный сплайсинг, глубокая интронная мутация

## Abstract

Парциальная форма синдрома резистентности к андрогенам (СРА) является наиболее сложным вариантом нарушения формирования пола 46,XY (НФП 46,XY) в плане выбора тактики ведения пациента. До настоящего времени не существует четких биохимических критериев, особенно в допубертатном возрасте, позволяющих дифференцировать данное заболевание от других форм НФП 46,XY, и молекулярно-генетическая диагностика парциальной формы СРА играет решающую роль. Между тем, по данным литературы, более чем у 50% пациентов с подозрением на СРА мутации в кодирующей области AR выявить не удается. Нами проведен расширенный анализ гена AR у пациента с клинико-­лабораторными признаками СРА и обнаружена глубокая интронная мутация в гене AR (с.2450–42G>A). Данная замена привела к формированию альтернативного сайта сплайсинга и, как следствие, нарушению функции AR. Описанный случай свидетельствует о необходимости углубленного генетического анализа в когорте пациентов с подозрением на СРА при отсутствии у них мутаций в гене AR при использовании стандартных методов молекулярно-генетической диагностики.

## Abstract (en)

Partial androgen resistance syndrome (PAIS) is the most difficult form of disorders/differences of sex development 46,XY (DSD 46,XY) for choosing of patient management. To date, there are no clear biochemical criteria, especially before puberty, that allow differentiating PAIS from other PAIS-like forms of DSD 46, XY, and genetic verification of the partial form of AIS plays an important role. Meanwhile, according to the literature, mutations in the coding region of AR gene have not been identified in more than 50% of patients with suspected AIS. We performed an extensive analysis of the AR gene in a patient with clinical and laboratory signs of AIS and found a deep intron mutation in the AR gene (p. 2450–42G>A). This variant creates an alternative splice acceptor site resulted a disturbance of the AR function. These findings indicate the need for extensive genetic analysis in a cohort of patients with suspected CPA in the absence of mutations in the AR gene using standard methods of genetic diagnosis.

## АКТУАЛЬНОСТЬ

Синдром резистентности к андрогенам (СРА) — это одна из наиболее распространенных форм нарушений формирования пола (НФП) 46,XY, обусловленная патологическими изменениями в гене рецептора к андрогенам (AR). Ген AR расположен на длинном плече хромосомы X (Xq11–12) и состоит из 8 экзонов. Заболевание является Х-сцепленным и проявляется только при кариотипе 46,XY, тогда как матери пациентов являются носителями мутации и не имеют никаких клинических проявлений. В зависимости от степени нарушения функции рецептора клинически выделяют полную, неполную, или парциальную, и мягкую формы СРА [1]. При полной форме заболевания фенотип ребенка при рождении правильный женский, в связи с чем вопрос о выборе пола воспитания обычно всегда решается в пользу женского. При мягкой форме заболевания фенотип правильный мужской, и диагноз нередко устанавливается только в период пубертата в связи с развитием гинекомастии или в период репродуктивного возраста в связи с бесплодием. Наиболее сложной в плане выбора пола воспитания является парциальная форма СРА, при которой степень нарушения строения наружных половых органов значительно варьирует от незначительной гипертрофии клитора до стволовой формы гипоспадии, что требует проведения дифференциальной диагностики с другими формами НФП 46,XY. Подтверждение парциальной формы СРА при низкой степени маскулинизации наружных половых органов играет ключевую роль в выборе пола воспитания ребенка, который при данной форме заболевания предпочтителен в пользу женского, с учетом невозможности достижения адекватной маскулинизации, несмотря на проведение гормональной терапии андрогенами в супрафизиологических дозах [2]. Заподозрить парциальную форму СРА можно на основании клинико-лабораторных признаков заболевания, но окончательно диагноз подтверждается молекулярно-генетически. Между тем у части пациентов с клинико-лабораторными проявлениями СРА мутации в гене AR не выявляются (до 25–60%) [3][4], что оставляет неопределенность в окончательной верификации диагноза, а следовательно, и трудности в выборе пола воспитания ребенка.

Ниже мы описываем случай парциальной формы СРА, обусловленной глубокой интронной мутацией в гене AR (с.245–42G>A), приведшей к формированию альтернативного сайта сплайсинга.

## ОПИСАНИЕ СЛУЧАЯ

Пациент М.: при рождении диагностирована мошоночная форма гипоспадии, двусторонний паховый крипторхизм, в ходе цитогенетического исследования установлен кариотип 46,XY, на основании чего пациент зарегистрирован в мужском паспортном поле. Из анамнеза известно, что брак родителей не близкородственный, семейный анамнез отягощен (дядя по материнской линии оперирован по поводу гипоспадии и гинекомастии) (рис. 1). В допубертатном возрасте проведены оперативные вмешательства: двусторонняя орхипексия и пластика уретры в 3 этапа. С 11-летнего возраста отмечено прогрессирующее развитие грудных желез, перераспределение подкожно-жировой клетчатки по феминному типу. При обследовании в возрасте 13 лет: половое развитие по Таннеру: G2Р2B3, половой член искривлен, длина до 3 см, кавернозные тела развиты слабо, тестикулы в мошонке, D=S ~6–8 мл, в гормональном профиле: лютеинизирующий гормон (ЛГ) — 5,0 Ед/л (2,5–11,0), фолликулостимулирующий гормон (ФСГ) — 0,9 Ед/л (1,55–9,74), тестостерон — 17,8 нмоль/л (мальчики II ст. по Таннеру 0,9–12,5), эстрадиол — 120 пмоль/л (мальчики II ст. по Таннеру 20–100). По данным ультразвукового исследования малого таза: дериваты мюллеровых протоков не визуализировались. При проведении пробы с хорионическим гонадотропином отмечено адекватное повышение андрогенов (тестостерона — с 17,8 до 35,2 нмоль/л, андростендиона — с 7,7 до 11,4 нмоль/л), что позволило исключить нарушение биосинтеза тестостерона как возможную причину НФП. На основании полученных данных диагностирован «синдром резистентности к андрогенам, парциальная форма». Учитывая мужской пол воспитания, проведена двусторонняя мастэктомия, к терапии добавлены блокаторы ароматазы.

Для верификации диагноза проведено секвенирование гена AR по Сэнгеру, однако патологических вариантных замен выявлено не было. При дополнительном молекулярно-генетическом анализе методом высокопроизводительного параллельного секвенирования (IonTorrent) с использованием таргетной панели «Нарушения формирования пола 46,XY» (43 гена) патологически значимых изменений также выявлено не было.

Учитывая клинико-лабораторные данные, свидетельствующие о наличии парциальной формы СРА при отсутствии изменений в каких-либо других генах-кандидатах, ассоциированных с НФП 46,XY, предположено наличие варианта нуклеотидной последовательности, влияющего на сплайсинг. Подобные замены по аналогии с другими заболеваниями [5] могут располагаться глубоко в интронах и не выявляться при секвенировании экзонов и экзон-интронных стыков методами Сэнгера или NGS. Для поиска возможных нарушений сплайсинга была выделена общая РНК из фибробластов кожи мошонки пациента и синтезирована библиотека кДНК. Используя кДНК в качестве матрицы, анализ специфических для гена AR последовательностей выявил наличие транскрипта дикого типа (NM_000044.3), а также аберрантный транскрипт, содержащий вставку из 40 нуклеотидных оснований (с.2450_2451insc.251–40_c.251–1), приводящий к сдвигу рамки считывания и образованию преждевременного стоп-кодона в позиции 842 (рис. 2). Дополнительное секвенирование последовательности интрона 6 гена AR с использованием геномной ДНК выявило гемизиготную транзицию hg38_chrX:67722785G>A, располагающуюся в интроне на дистанции в 42 нуклеотидных основания перед началом экзона 7 (с.2450–42 G>A) (рис. 3).

**Figure:**
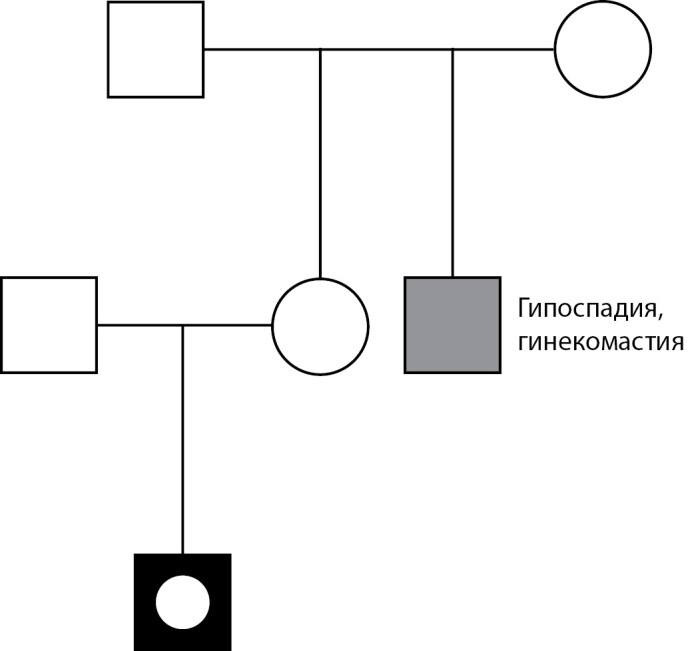
Рисунок 1. Родословная пациента

**Figure:**
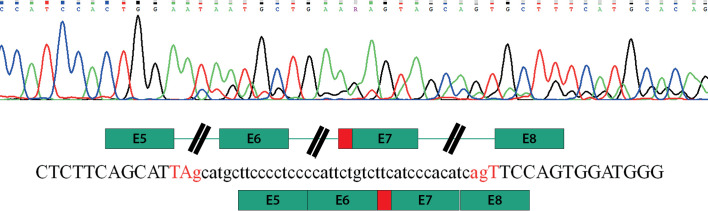
Рисунок 2. Аберрантный сплайсинг при секвенировании кДНК из фибробластов кожи мошонки.

**Figure:**
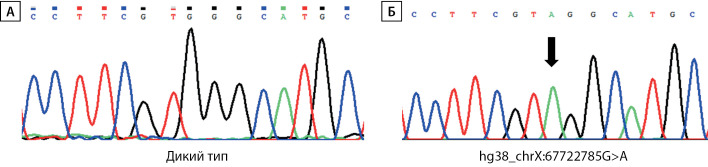
Рисунок 3. Секвенирование интрона 6 гена AR с использованием геномной ДНК.А — фрагмент геномной последовательности дикого типа в интроне 6 (hg38_chrX:67722778-67722792); Б — гемизиготная транзиция hg38_chrX:67722785G>A (указано стрелкой), располагающаяся в интроне 6, на дистанции в 42 нуклеотидных основания перед началом экзона 7 (с.2450-42 G>A)

## ОБСУЖДЕНИЕ

J. Morris в 1953 г. впервые опубликовал описание серии случаев СРА (80 случаев из литературы и 2 собственных наблюдения), когда у пациентов при женском фенотипе имелись тестикулы [6], и ввел термин «синдром тестикулярной феминизации». Однако еще в 1950 г. L. Wilkins предположил патофизиологическую основу СРА, отметив у наблюдаемого им пациента с женским фенотипом и тестикулами («безволосая женщина с яичками») нормальную экскрецию андрогенов и эстрогенов в моче и отсутствие маскулинизации в ответ на лечение 50 мг метилтестостерона. На основании полученных данных Wilkins сделал вывод о наличии резистентности органов-мишеней к андрогенам [7]. Клонирование кДНК рецептора к андрогенам человека в 1988 г. позволило T. Brown и соавт. подтвердить, что в основе СРА действительно лежат патологические изменения в гене AR[8]. В последующем, благодаря широкому внедрению полимеразной цепной реакции и секвенирования по Сэнгеру, анализ кодирующей последовательности гена AR стал неотъемлемой частью подтверждающей диагностики СРА [9].

К настоящему времени описано более 550 точковых патологических вариантов нуклеотидных последовательностей, приводящих к замене аминокислоты, делеции/вставки и сдвигу рамки считывания с образованием преждевременного стоп-кодона [http://www.hgmd.cf.ac.uk/ac/index.php]. Между тем, как отмечают некоторые авторы, несмотря на наличие у пациентов четких клинико-лабораторных данных, предполагающих СРА, идентифицировать мутации при секвенировании кодирующей области гена AR удается не всегда: в ~95% случаев при полной форме и менее чем в 40% случаев при парциальной форме заболевания [3][10].

В литературе имеются единичные описания СРА вследствие глубоких интронных мутаций, для которых доказано влияние на сплайсинг, и обнаружение которых при стандартном методе анализа гена AR невозможно [1][11–13]. Так, в 1990 г. Rip и соавт. [11] описали у пациента с полной формой СРА наличие делеции >6 kb во втором интроне, которая затрагивала консервативную последовательность ответвления (branch site), что привело к отсутствию в транcкрипте экзона 3 и сдвигу рамки считывания, при этом около 90% всей мРНК составлял аберрантный вариант и лишь 10% — дикий тип. H. Brüggenwirth и соавт. выявили патологическую замену T>A в интроне 2 в позиции c.1769–11, приведшую к формированию альтернативного сайта сплайсинга. Трансляция аберрантного транскрипта приводила к появлению белка AR с дополнительными 23 аминокислотными остатками, вставленными между двумя цинковыми пальцами, полностью нарушив связывание AR с ДНК, и, как следствие, к развитию полной формы СРА [13]. J. Känsäkoski и соавт. описали случай полной формы СРА, обусловленный глубокой интронной мутацией в интроне 6 (c.2450–118A>G), приведшей к образованию двух транскриптов с преимущественным синтезом аберрантного и отсутствию экспрессии AR в фибробластах пациента [12].

Обращает на себя внимание, что все описанные выше случаи СРА, обусловленные нарушением сплайсинга вследствие глубоких интронных мутаций, приводили к преимущественной экспрессии аберрантного транскрипта, что клинически проявилось развитием полной формы СРА. В нашем случае результаты секвенирования кДНК свидетельствуют о преимущественной экспрессии транскрипта дикого типа, что может объяснять развитие парциальной формы СРА.

Важно отметить, что, несмотря на редкость описаний глубоких интронных мутаций как причины возникновения СРА, в 2018 г. H. Ono и соавт. при обследовании семьи с парциальной формой СРА в двух поколениях выявили вариант нуклеотидной последовательности в той же позиции, что и в представляемом нами случае. Авторами также была показана преимущественная экспрессия транскрипта дикого типа в сравнении с мутантным [14]. Таким образом, наличие идентичной гемизиготной замены с.2450–42 G>A в глубокой области интрона 6 гена AR у неродственных пациентов с клинической картиной парциальной формы СРА подтверждает ее патогенность и свидетельствует о схожем механизме нарушения функции рецептора к андрогенам.

Представленное нами наблюдение и данные литературы свидетельствуют об ограничениях стандартного подхода к молекулярно-генетическому анализу гена AR, при котором проводится секвенирование экзонов и участков интронов в области экзон-интронных стыков. Учитывая, что геномная протяженность гена AR составляет более 186 500 пар нуклеотидных оснований, которые преимущественно занимают интроны, и лишь 2763 (~1,5%) соответствуют кодирующей последовательности, вероятность наличия патогенных мутаций глубоко в интронах достаточно высока. Анализ гена AR является основополагающим в диагностике СРА, от результатов которого при парциальной форме заболевания зависит выбор пола воспитания ребенка. Между тем «отрицательный» результат по данным стандартного молекулярно-генетического исследования не может полностью исключить нарушение функции AR. Анализ транскриптов AR, экспрессируемых в фибробластах в доступных андрогензависимых участках кожи, может быть ценным дополнительным методом обследования, который будет способствовать улучшению качества диагностики при НФП 46,XY.

## ЗАКЛЮЧЕНИЕ

Исходя из вышеописанного, можно предположить, что использование более сложных методов молекулярно-генетической диагностики при дообследовании пациентов с клинико-лабораторными данными СРА, но не имеющих генетического подтверждения в ходе использования стандартных методов молекулярно-генетической диагностики позволит выявить большее число глубоких интронных вариантных замен нуклеотидных последовательностей, приводящих к СРА. Возможно, выявленный нами вариант транзиции G в A, являясь более частым, чем трансверзии, может оказаться «горячей» точкой, а не редким вариантом причины СРА.

## ДОПОЛНИТЕЛЬНАЯ ИНФОРМАЦИЯ

Источники финансирования. Работа выполнена при содействии Фонда поддержки и развития филантропии «КАФ».

Конфликт интересов. Авторы декларируют отсутствие явных и потенциальных конфликтов интересов, связанных с содержанием настоящей статьи.

Участие авторов. Калинченко Н.Ю. — концепция и дизайн исследования, сбор материала, анализ данных, написание текста; Петров В.М. — анализ данных, интерпретация результатов; Панова А.В. — анализ данных, интерпретация результатов; Тюльпаков А.Н. — концепция и дизайн исследования, интерпретация результатов, внесение в рукопись существенной правки с целью повышения научной ценности статьи. Все авторы одобрили финальную версию статьи перед публикацией, выразили согласие нести ответственность за все аспекты работы, подразумевающую надлежащее изучение и решение вопросов, связанных с точностью или добросовестностью любой части работы.

Согласие пациента. Добровольное информированное согласие пациента на публикацию в журнале «Проблемы эндокринологии» получено.

Благодарности. Выражаем благодарность Фонду поддержки и развития филантропии «КАФ» за помощь в проведении исследования.
